# Supplementary figures and images for: Outreach screening to address demographic and economic barriers to diabetic retinopathy care in rural China
Source: PLoS One. 2022 Apr 20;17(4):e0266380. doi: 10.1371/journal.pone.0266380 (PMC9020743; doi:10.1371/journal.pone.0266380)

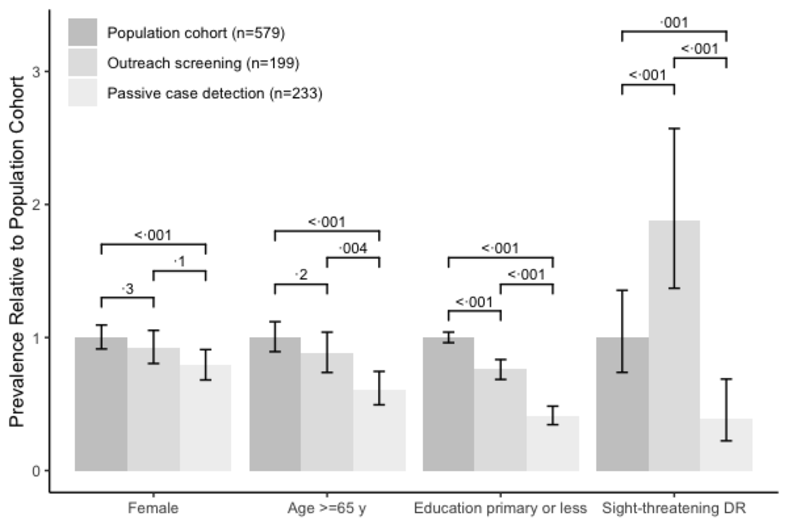

Supplement: S1 Fig — (TIF) [file pone.0266380.s001.tif]
